# A new look at the feeding behaviour of haematophagous Diptera: the biting habits of *Leptoconops (Megaconops) floridensis* Wirth (Diptera: Ceratopogonidae)

**DOI:** 10.1590/0074-02760260021

**Published:** 2026-09-28

**Authors:** Giulia Gaglianone Lemos, Jenyffer Vierheller Vieira, Natacha Zimmermann dos Santos, Danilo Leite Sforcin, Thiago Felipe de Souza, Aurélio Gonçales Bezerra, Manoela LP Miranda, Anna Kelly Krassota, Maria Clara Alves Santarém, Maria Luiza Felippe-Bauer, Marta Jussara Cremer

**Affiliations:** 1 Universidade da Região de Joinville, Centro de Reabilitação de Fauna Marinha, São Francisco do Sul, SC, Brasil; 2 Universidade da Região de Joinville, Laboratório de Ecologia e Conservação de Tetrápodes Marinhos e Costeiros, São Francisco do Sul, SC, Brasil; 3 Fundação Oswaldo Cruz-Fiocruz, Instituto Oswaldo Cruz, Laboratório de Diptera, Coleção de Ceratopogonidae, Rio de Janeiro, RJ, Brasil

**Keywords:** animal health, biting midges, birds, haematophagy, Brazil

## Abstract

**BACKGROUND:**

Understanding ecological relationships among species and their pathogens is essential for assessing disease dynamics and associated risks to human, animal, and environmental health. Some haematophagous ceratopogonid species feed on birds and may act as vectors for haemoparasites, viruses, and bacteria. However, no reports have documented these insects feeding within the internal organs of birds.

**OBJECTIVES:**

This study aims to describe the first recorded occurrence of ceratopogonids feeding within the respiratory system of debilitated kelp gulls and cormorants.

**METHODS:**

As part of the Beach Monitoring Project of the Santos Basin (PMP-BS Area SC/PR - IBAMA - ABIO No. 640/2015), we rescued debilitated coastal birds from the municipalities of São Francisco do Sul and Itapoá, in northern Santa Catarina State, Brazil, and examined the biting midges infesting them.

**FINDINGS:**

Approximately 18.2% (31/172) of the kelp gulls and 15.8% (3/19) of the cormorants analysed were infested internally with *Leptoconops* (*Megaconops*) *floridensis* Wirth and, in sporadic cases, with two species of *Culicoides*. Kelp gulls accounted for 91.2% of the infested birds (31/34), and the trachea, air sacs, and lungs were the most frequently affected regions of the respiratory system (~83.9%).

**MAIN CONCLUSIONS:**

This is the first recorded occurrence of ceratopogonids feeding within the innermost part of the avian respiratory system.

Birds commonly host a wide range of ectoparasites, including chewing lice, sucking lice, mites, ticks, fleas, and flies.^1^ [Bibr B2] ^2^ However, no records have documented these taxa as endoparasites. Numerous parasites have been reported in the respiratory system of birds, including the nasal sinuses, trachea, and air sacs. Among them are nematodes (*Syngamus* spp., *Cyathostoma* spp., and *Serratospiculum* spp.), pentastomids (*Reighardia sternae*), mites (*Sternostoma tracheacolum*), and trematodes (*Tracheophilus* spp.).^3^

The avian respiratory system is unique and widely considered the most efficient among vertebrates. It comprises a parabronchial lung of constant volume connected to a system of nine air sacs. Although essential for ventilation, the air sacs do not participate in gas exchange; they consist of thin, transparent, poorly vascularised membranes attached to the walls of the coelomic cavity and expand and contract through sternal and rib movements, driving air in and out of the parabronchial lung.^4^ [Bibr B5] ^5^ [Bibr B6] ^6^

The generalist, opportunistic foraging habits of many seabird species make them particularly vulnerable to anthropogenic impacts, which are often more intense in coastal zones. Consequently, birds may become debilitated and strand on beaches due to intoxication, fishery-related injuries, trauma, and other causes.^7^ [Bibr B8] ^8^ *Larus dominicanus* (Lichtenstein, 1823), commonly known as the kelp gull, is one of the most common and abundant seabird species along the southeastern and southern coasts of Brazil.^9^ It occupies a variety of coastal habitats, including tidal flats, beaches, and rocky shores, as well as urbanised environments and waste disposal sites.^10^ [Bibr B11] ^11^ [Bibr B12] ^12^ The species exhibits generalist, opportunistic feeding behaviour, functioning as a predator, scavenger, and kleptoparasite.^13^ [Bibr B14] ^14^ [Bibr B15] ^15^ [Bibr B16] ^16^

The neotropic cormorant (*Nannopterum brasilianum* Gmelin, 1789) is widely distributed throughout Brazil and occupies a broad range of wetland habitats, including estuaries, beaches, lagoons, rivers, reservoirs, ponds, high-altitude lakes, and other freshwater environments.^15^ [Bibr B17] ^17^ [Bibr B18] ^18^ In Santa Catarina State, it is considered abundant, and some breeding sites have been documented there.^10^ [Bibr B19] ^19^ Studies on the feeding ecology of the neotropic cormorant indicate that its diet consists primarily of bony fishes (Actinopterygii), crustaceans, molluscs, and amphibians.^20^ [Bibr B21] ^21^ [Bibr B22] ^22^ Nevertheless, the species displays considerable adaptability, responding effectively to variation in available environmental resources.^22^

Haematophagous arthropods belonging to the families Culicidae, Simuliidae, and Ceratopogonidae, and to the subfamily Phlebotominae, have been reported feeding on wild birds worldwide. These insects are considered potential vectors for haemoparasites, including protozoans of the genera *Haemoproteus* Kruse, *Plasmodium* Marchiafava & Celli and *Leucocytozoon* Sambon.^1^ [Bibr B3] ^3^ Within dipterans of the family Ceratopogonidae, species of the genus *Culicoides* Latreille, commonly known as biting midges, are important vectors of numerous parasites (*e.g.*, filarial worms, bacteria, protozoans, and viruses), posing a threat to human, domestic animal, and wildlife health.^23^

The cosmopolitan genus *Leptoconops* is distributed in humid areas with sandy or clayey soils, particularly in coastal marshes and saltwater lagoons.^24^ Due to the diurnal activity of its species in various coastal regions, the genus has been regarded as a pest, negatively affecting beach tourism. Little is known about the taxonomy and bionomics of this genus; accordingly, only three species have been recorded in Brazil: *Leptoconops (Leptoconops) brasiliensis* (Lutz),^25^ in Acre, Amazonas, Pará, and Goiás; *Leptoconops (H.) knowltoni* Clastrier & Wirth,^26^ in Santa Catarina;^27^ and, more recently, *Leptoconops (Megaconops) floridensis* Wirth, also in Santa Catarina.^28^

We report a novel ecological interaction involving haematophagous dipterans infesting the respiratory system of kelp gulls and neotropic cormorants rescued in debilitated condition in coastal environments of northern Santa Catarina State, southern Brazil.

## MATERIALS AND METHODS

*Study area* - The study area comprises the northern coast of Santa Catarina State (southern Brazil), extending from the northern boundary of the municipality of Itapoá (25º58'34.8" S/48º35'46.1" W) to the southern boundary of the municipality of Araquari (26º34'49.8" S/48º39'50.5" W), including the municipalities of São Francisco do Sul and Balneário Barra do Sul, as well as the Babitonga Bay estuary (Fig. 1). The region has a humid subtropical climate, with an average annual precipitation ranging from 1,700 to 2,900 mm. Summer is the wettest season (600-900 mm), while winter is the driest (300-400 mm). The mean annual temperature is 22.41ºC,^29^ with the coldest month averaging around 16ºC. The highest temperatures occur between November and March, occasionally exceeding 30ºC.^30^

**Fig. 1:**
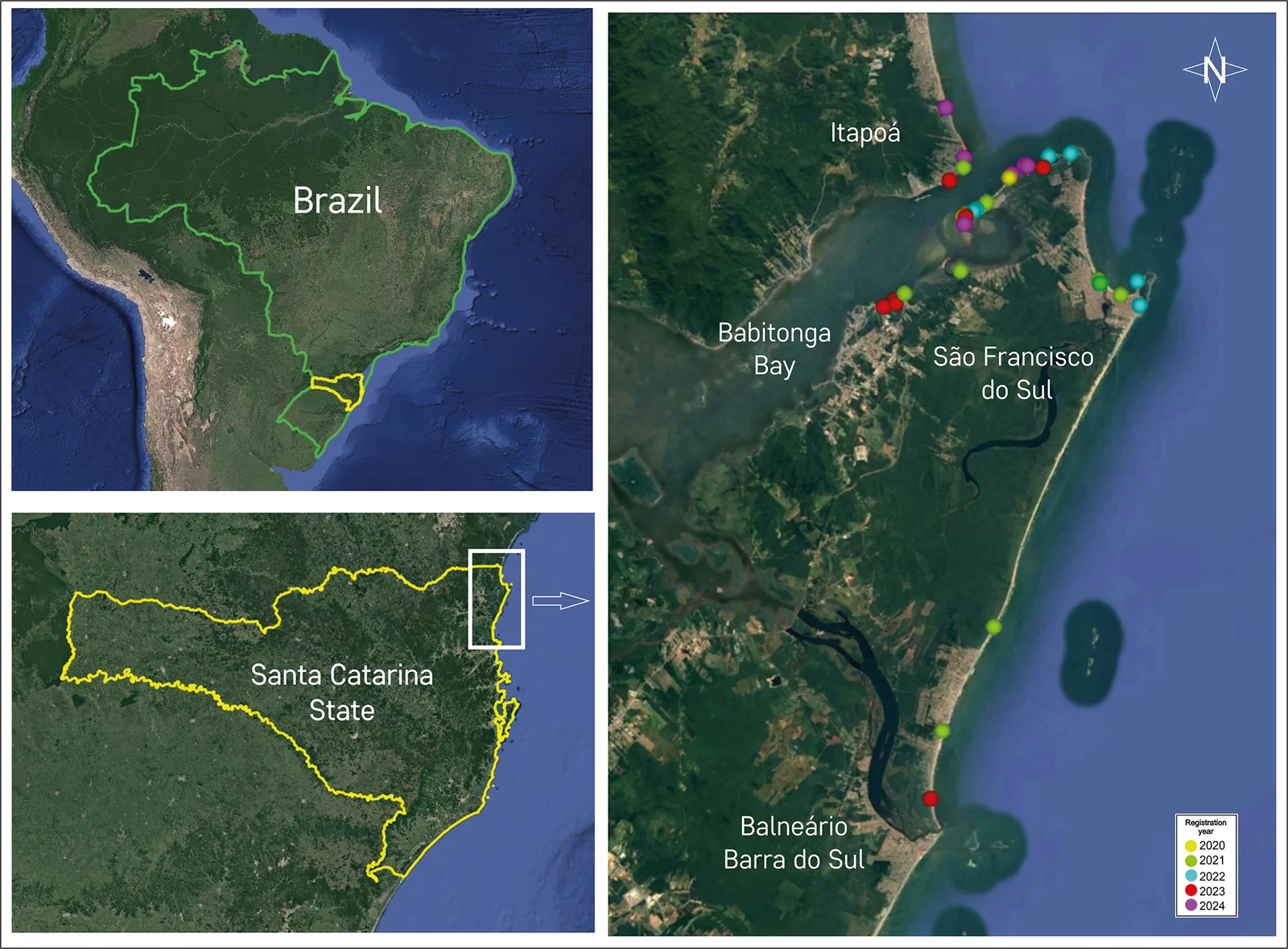
study area (northern Santa Catarina State, southern Brazil) and the sites where the debilitated kelp gulls (*Larus dominicanus*) and neotropic cormorants (*Nannopterum brasilianum*) analysed in this study were recorded.

The region harbours a significant diversity of ecosystems within the Atlantic Forest biome, such as mangroves, coastal sand dune vegetation (restingas), and ombrophilous forests.^31^ The main beaches where the birds analysed in this study were recorded exhibit two distinct granulometric and morphodynamic domains: (1) a predominance of medium sand with an intermediate to reflective morphodynamic state, associated with more exposed beaches; and (2) fine sand with dissipative to intermediate behaviour, on more sheltered, semi-exposed beaches. The region also includes beaches under the estuarine influence of Babitonga Bay, located along the access channel and throughout the estuary.^32^

*Monitoring strategy of marine tetrapods* - The northern coast of Santa Catarina has been monitored daily to record dead and debilitated live marine tetrapods, as part of the Beach Monitoring Project of the Santos Basin (*Projeto de Monitoramento de Praias da Bacia de Santos* — PMP-BS), since August 2015. This initiative fulfils a requirement of the federal environmental licensing process for oil and gas exploration and production activities in the Santos Basin, conducted by the Brazilian Institute of Environment and Renewable Natural Resources (*Instituto Brasileiro do Meio Ambiente e dos Recursos Naturais Renováveis* — IBAMA). For this study, we considered live debilitated birds recorded between December 2020 and February 2024, starting with the first recorded case of Ceratopogonidae in the respiratory system of this group, in December 2020. Only debilitated live birds were considered, including individuals that died during treatment; birds found dead were excluded from the analysis to avoid potential postmortem bias.

Debilitated birds were rescued and referred to the *Centro de Reabilitação de Fauna Marinha* (Univille), located in São Francisco do Sul [Wildlife Management Authorisation No. 1073970, issued by the Santa Catarina Environmental Institute (*Instituto do Meio Ambiente de Santa Catarina* — IMA)], where they received appropriate veterinary care according to the specific requirements of each species. Throughout the treatment period, the birds were housed in screened enclosures, isolated from the external environment. The presence of ceratopogonids was documented in some birds during the initial physical examination or during routine handling for medication, hydration, and feeding. On these occasions, insects that emerged alive, often flying out from the nares, choanae, and glottis, were collected when possible. When birds exhibited poor health conditions deemed irreversible upon veterinary evaluation, euthanasia was performed following the guidelines of the Brazilian Manual of Good Practices for Euthanasia (*Guia Brasileiro de Boas Práticas em Eutanásia em Animais — Conceitos e Procedimentos Recomendados*).^33^ A detailed necropsy was conducted for these individuals and for those that died during treatment. During these procedures, both live and dead insects were collected. Notably, the number of live insects was likely underestimated, as some escaped through the oral cavity while the hosts were still alive, and in some cases during the opening of the coelomic cavity at necropsy.

Body condition was scored on a four-stage scale (optimal, good, thin, or emaciated) based on keel palpation, assessment of pectoral musculature, and subcutaneous and visceral fat content.^34^ Sex was determined only for birds that died and were necropsied. Developmental stage was determined by macroscopic analysis of the gonads at necropsy or, in live birds, solely by plumage pattern. On admission, patients were assessed by a specialised veterinarian, including physical examination, blood tests, faecal exams, and radiography to guide diagnosis and treatment. Disorders diagnosed antemortem and postmortem — in addition to biting midge infestation — are referred to in this study as "associated disorders" and were considered potential risk factors for the birds' exposure to, and infestation by, the insects.

*Ethics statement* - Ethical approval was not required for this study, as all patients were managed exclusively within the scope of routine veterinary care.

The sites where the infested birds were rescued were grouped into three categories: (1) outflow beaches, within the estuary domain; (2) semi-exposed/exposed beaches; and (3) estuary. This classification was based on geographic location and degree of wave exposure (*in situ* observations), as well as on the criteria established by Abreu.^32^ Laboratory procedures followed the protocols established by the PMP-BS and complied with the legal requirements of IBAMA (ABIO No. 640/2015).

*Analysis of biting midges* - The insects were manually collected, stored in 70% ethanol, and catalogued by the anatomical region of the bird from which they were obtained. They were subsequently transported to the Ceratopogonidae Collection, Diptera Laboratory, Fundação Oswaldo Cruz, Rio de Janeiro, where the morphotypes found were mounted on microscope slides in phenol-Canada balsam following the method described by Wirth and Marston.^35^ Data on Brazilian biodiversity generated by this study are registered with the National System for the Management of Genetic Heritage and Associated Traditional Knowledge (*Sistema Nacional de Gestão do Patrimônio Genético e do Conhecimento Tradicional Associado* — SisGen) under No. AA0BE39.

## RESULTS

From December 2020 to February 2024, 539 debilitated live birds of 26 species were rescued in the study area and treated under the PMP-BS. Of these, 6.3% (n = 34) exhibited ceratopogonids in the respiratory system, including 31 kelp gulls (31/172) and three neotropic cormorants (3/19). The infested birds were found on beaches in two municipalities, with a higher proportion in São Francisco do Sul (85%; 29/34) than in Itapoá (15%; 5/34). Most occurrences were at the mouth of Babitonga Bay, under the influence of the estuary (62%; 21/34), followed by semi-exposed/exposed sandy beaches (23%; 8/34), with some records in the inner part of the estuary (15%; 5/34) (Fig. 1).

*Kelp gull - L. dominicanus* - Infested kelp gulls were recorded over four consecutive spring/summer seasons and in the autumn of 2023 (Table). Among the infested birds, 9.6% (3/31) recovered, 58.1% (18/31) died naturally, and 32.3% (10/31) exhibited clinical conditions incompatible with rehabilitation and release into the wild, leading to euthanasia. Because of uncertainty about the life cycle of *Le. floridensis*, and even about its distribution in Brazil, the three kelp gulls confirmed to carry the biting midges in the respiratory system that fully recovered after treatment were released on the same beach where they were found, to prevent the insect from spreading to other regions of the Santa Catarina coast.

**TABLE:** 

Occurrence of infested birds per season	Nº	Sex (F/M/UN)	Developmental stage (J/A/UN)	Body condition score (E/T/G)
Spring/summer (2020/2021)				
*Larus dominicanus*	8	0/8/0	1/7/0	3/2/3
Spring/summer (2021/2022)				
*L. dominicanus*	7	3/3/1	4/3/0	4/2/1
*Nannopterum brasilianum*	3	1/2/0	0/3/0	2/0/1
Spring/summer (2022/2023)				
*L. dominicanus*	4	1/3/0	1/3/0	2/0/2
Autumn 2023				
*L. dominicanus*	2	0/2/0	0/2/0	1/0/1
Spring/summer (2023/2024)				
*L. dominicanus*	10	2/6/2	4/6/0	3/3/4

Number of infested birds by season and classification by sex (F = female; M = male; UN = undetermined), developmental stage (J = juvenile; A = adult; UN = undetermined), and body condition score (E = emaciated; T = thin; G = good) of kelp gulls (*L. dominicanus*) and neotropic cormorants (*N. brasilianum*) from December 2020 to February 2024.

The most frequent associated disorders were respiratory infections (n = 12; 30.8%), musculoskeletal trauma (n = 9; 23.1%), intoxication (n = 5; 12.8%), and musculoskeletal infections such as septic arthritis (n = 5; 12.8%); less frequently, ocular trauma, cardiovascular infections, kidney injury, and drowning were recorded (n = 8; 20.5%). In some cases, more than one disorder was present. Among the infested kelp gulls, 22 were male (71%), six were female (19.4%), and sex could not be determined for three individuals (9.6%), as these animals recovered and were released. Regarding developmental stage, 21 (67.7%) were adults and 10 (32.3%) were juveniles; body condition was classified as thin or emaciated in 20 individuals (64.5%) (Table).

The ceratopogonids were primarily collected from the trachea (n = 12 patients; 38.7%), followed by the air sacs (n = 8 patients; 25.8%) and lungs (n = 6 patients; 19.4%); they were also observed in the bronchi (n = 1 patient; 3.2%) and in the naso- and oropharynx (n = 4 patients; 12.9%) (Fig. 2). Anatomical location was determined by the organ in which the insects were most abundant. In live insects, movement from one organ to another within the respiratory system was observed. In 17 individuals (54.8% of cases), insects were collected from more than one infestation site, and were found in all segments of the respiratory system in the three most severe cases (Fig. 3). In eight cases (25.8%), live insects were observed feeding on the host's blood, associated with localised inflammation spots consistent with bites. A video showing ceratopogonids in the respiratory system of kelp gulls is available in the Supplementary data (Video).

**Fig. 2:**
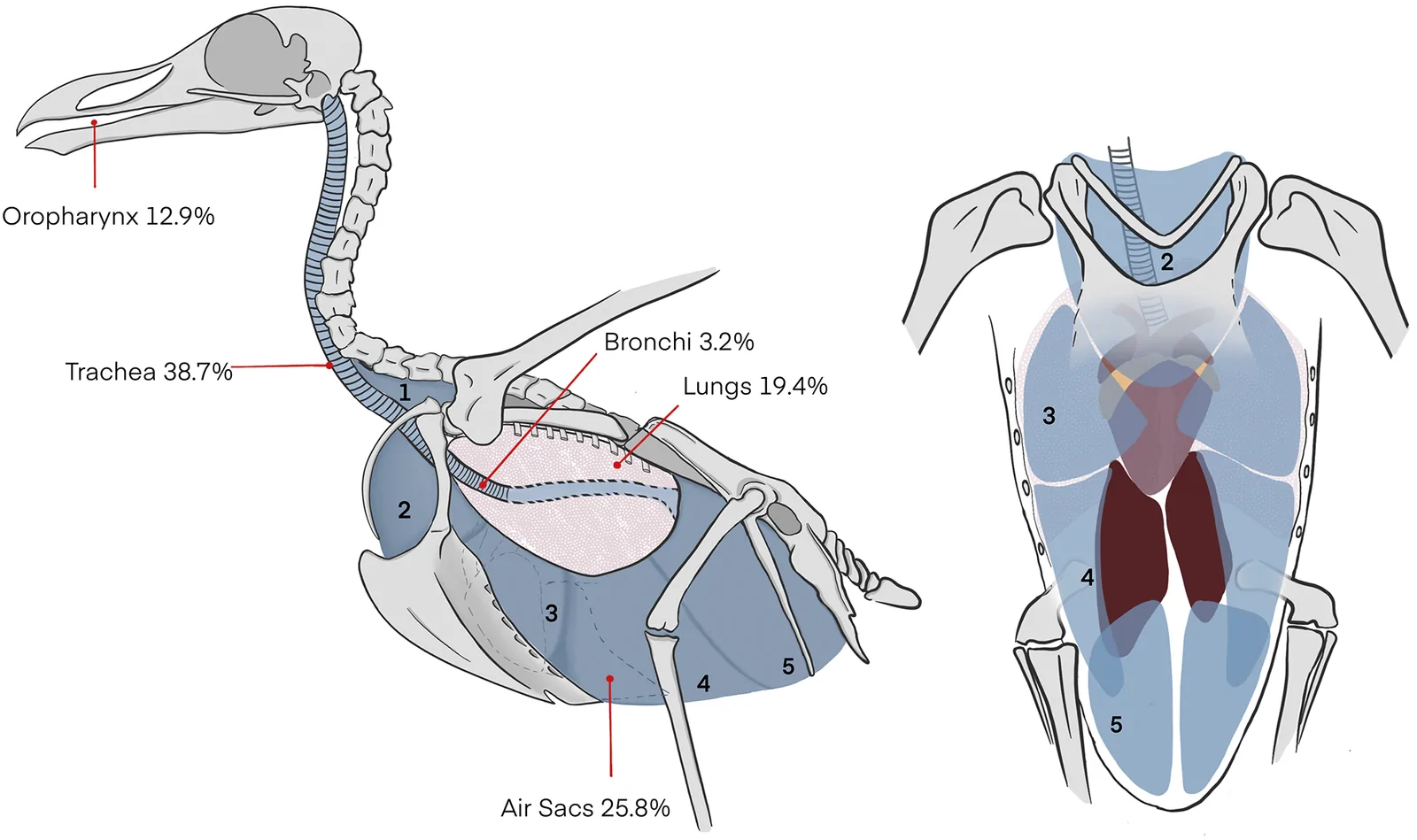
schematic diagram of the respiratory system of a kelp gull (*Larus dominicanus*), showing the percentage of ceratopogonid infestation in each region of the respiratory system. [Illustration by Giulia G. Lemos]. 1. Cervical air sacs; 2. Clavicular air sac; 3. Cranial thoracic air sacs; 4. Caudal thoracic air sacs; 5. Abdominal air sacs.

**Fig. 3:**
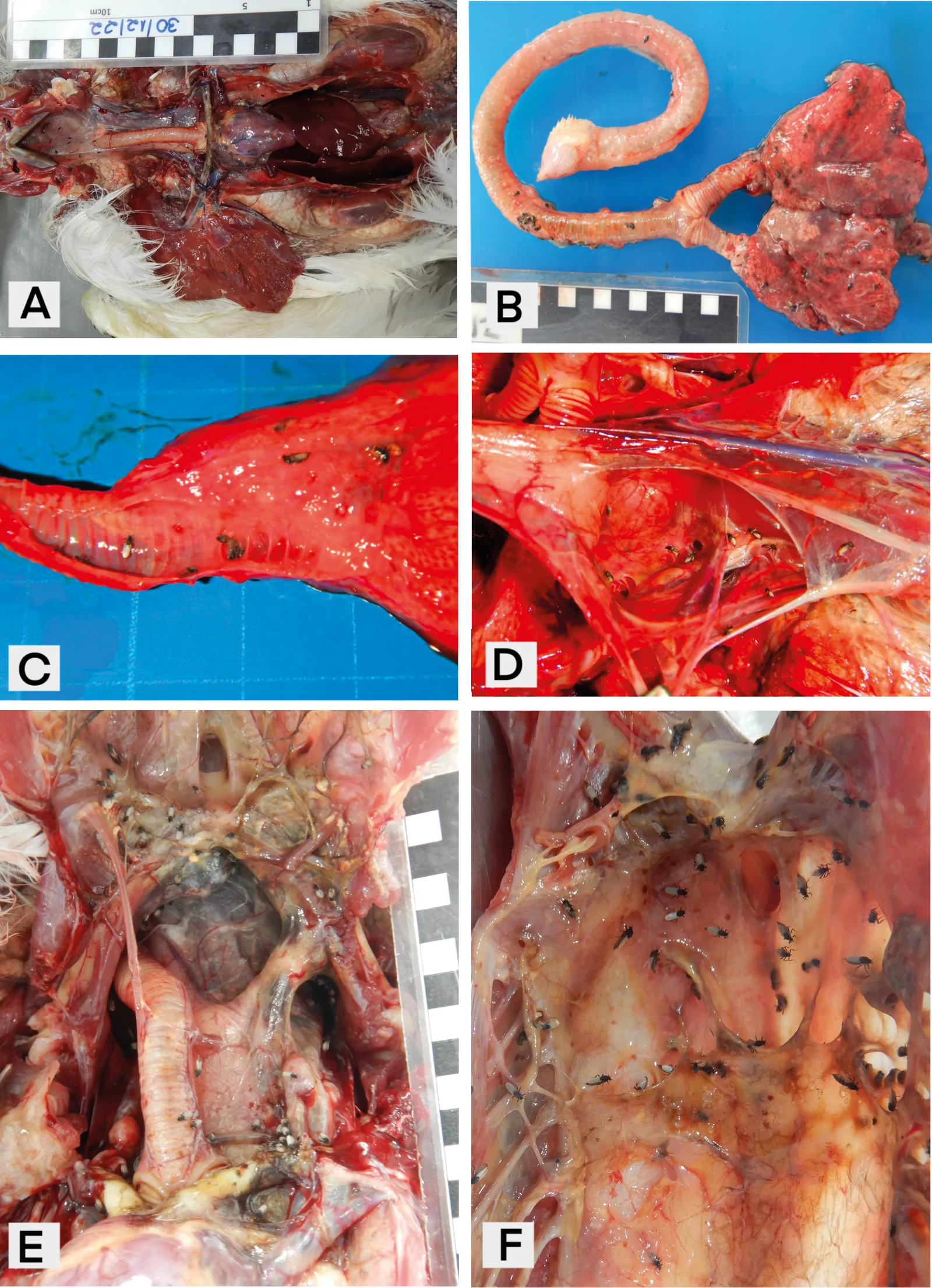
regions of the respiratory system of a kelp gull (*Larus dominicanus*) affected by biting midges (Ceratopogonidae). (A) Body cavity at necropsy; (B) Trachea, bronchi, and lungs; (C) Bronchi; (D) Cervical air sac; (E) Clavicular air sac, ventral view; (F) Clavicular air sac, sternum view.

Most ceratopogonids found in the lungs were dead, either encased in secretion or encapsulated (Fig. 4). Live insects were found predominantly in the air sacs. In five cases, kelp gulls were observed expelling live insects for up to five days after rescue. Insects were observed emerging from the glottis, nostrils, and choanae, generally preceded by bird vocalisations indicating respiratory discomfort. Although the insect count may have been underestimated, owing to the escape of live insects during collection, substantial variation in the degree of infestation was observed, ranging from one to 172 insects per bird. Excluding extreme values (> 51; n = 5), the mean was 4.5 ± 4.4 insects per bird.

**Fig. 4:**
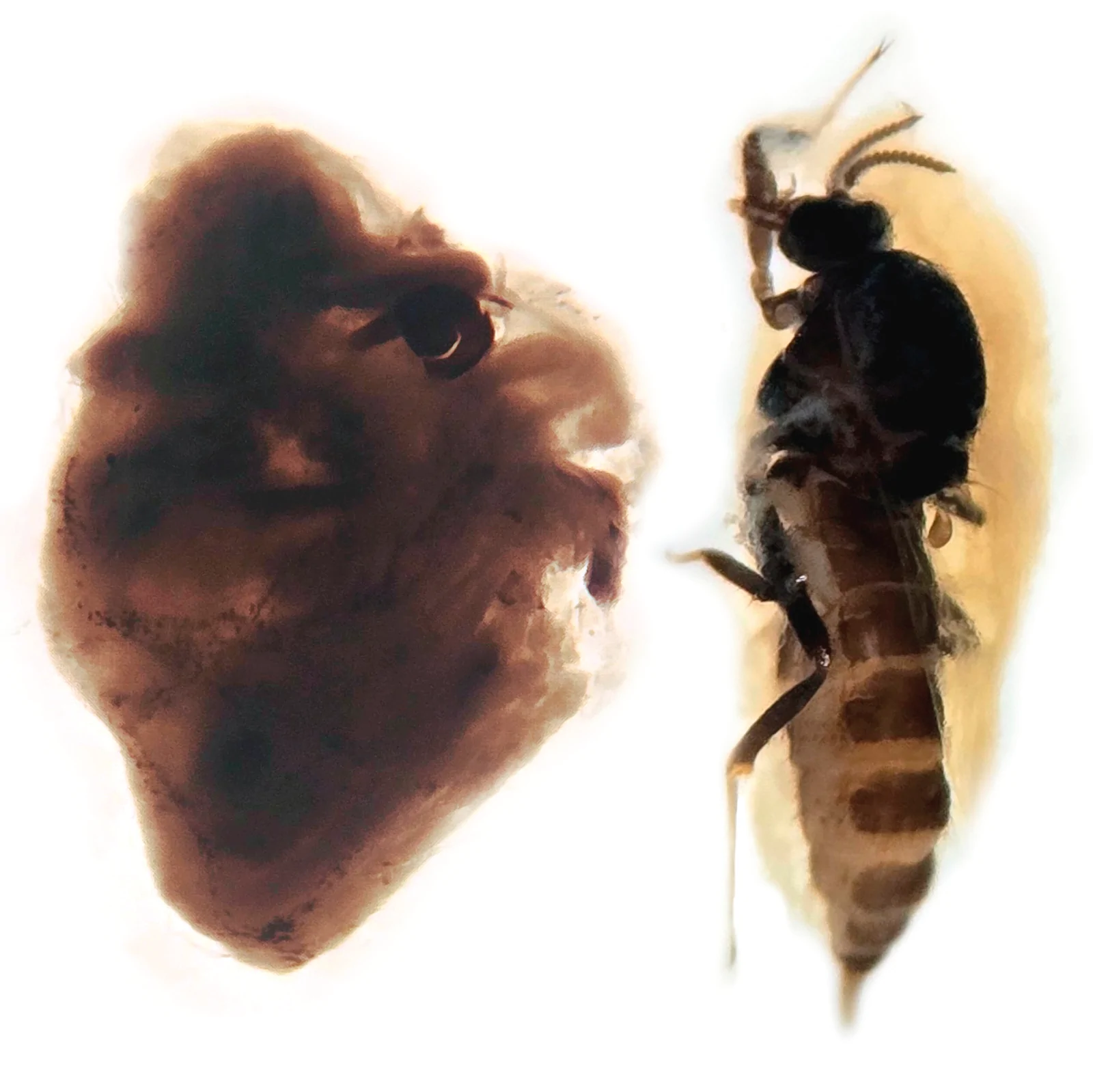
*Leptoconops* (*Megaconops*) *floridensis* female collected from the lung of a kelp gull (*Larus dominicanus*). Specimen encased in secretion and undergoing encapsulation (right to left).

*Neotropic cormorant - Nannopterum brasilianum* - Infestation was recorded in three animals rescued during the 2021/2022 spring and summer season. All three individuals were adults: one female and two males. Regarding body condition, two were emaciated and one had a good body score (Table). One individual died naturally of respiratory infection, and the other two were euthanised — one owing to severe musculoskeletal trauma and the other to severe neurological signs unresponsive to treatment. Live and dead insects were observed in the air sacs (n = 1 patient; 33.3%) and in the naso- and oropharynx (n = 2 patients; 66.7%). All infestations were mild, with an average of two insects per patient.

**Fig. 5:**
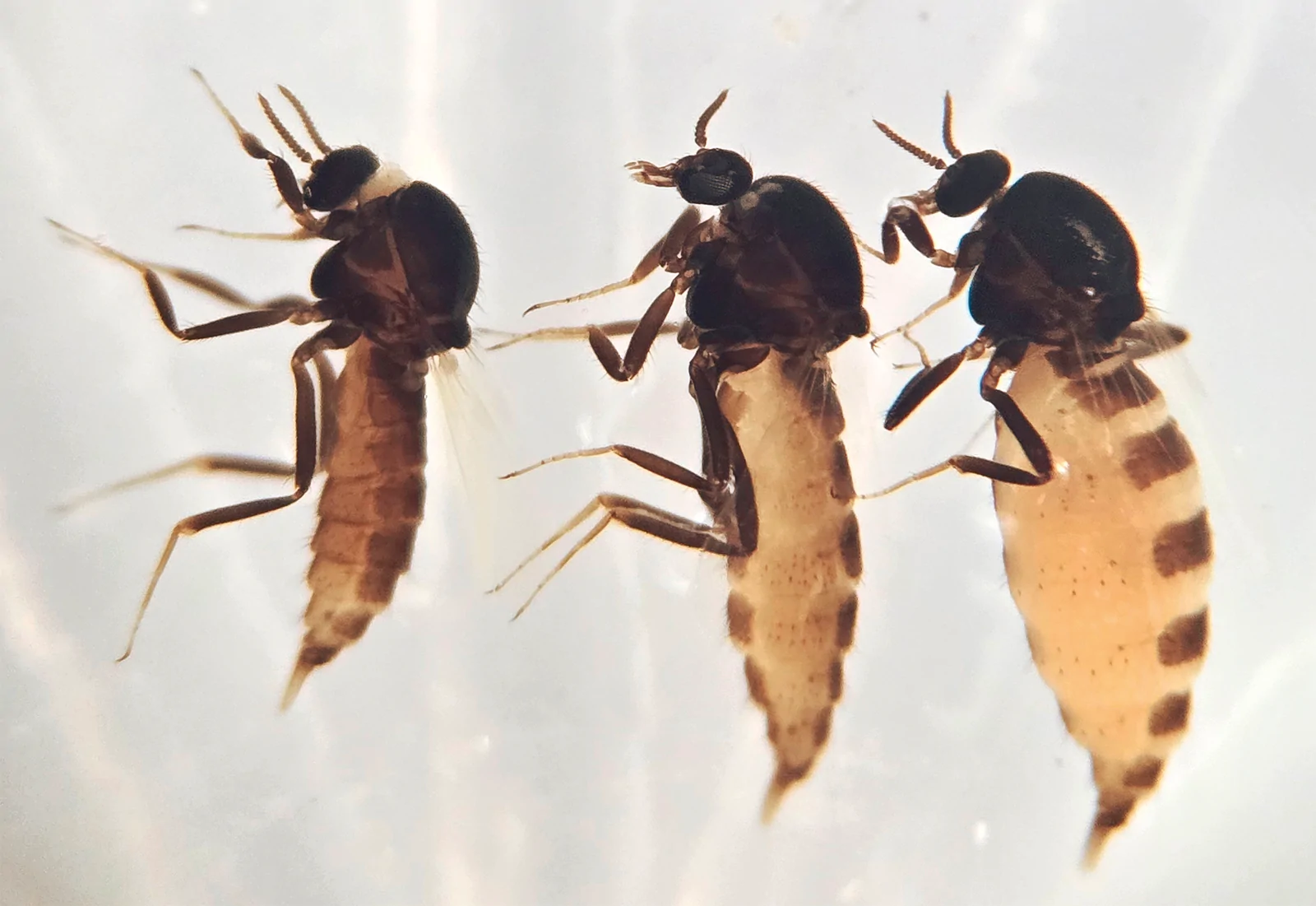
*Leptoconops* (*Megaconops*) *floridensis* collected from the lung of a kelp gull (*Larus dominicanus*). Females without blood meal, engorged, and gravid (left to right).

**Fig. 6:**
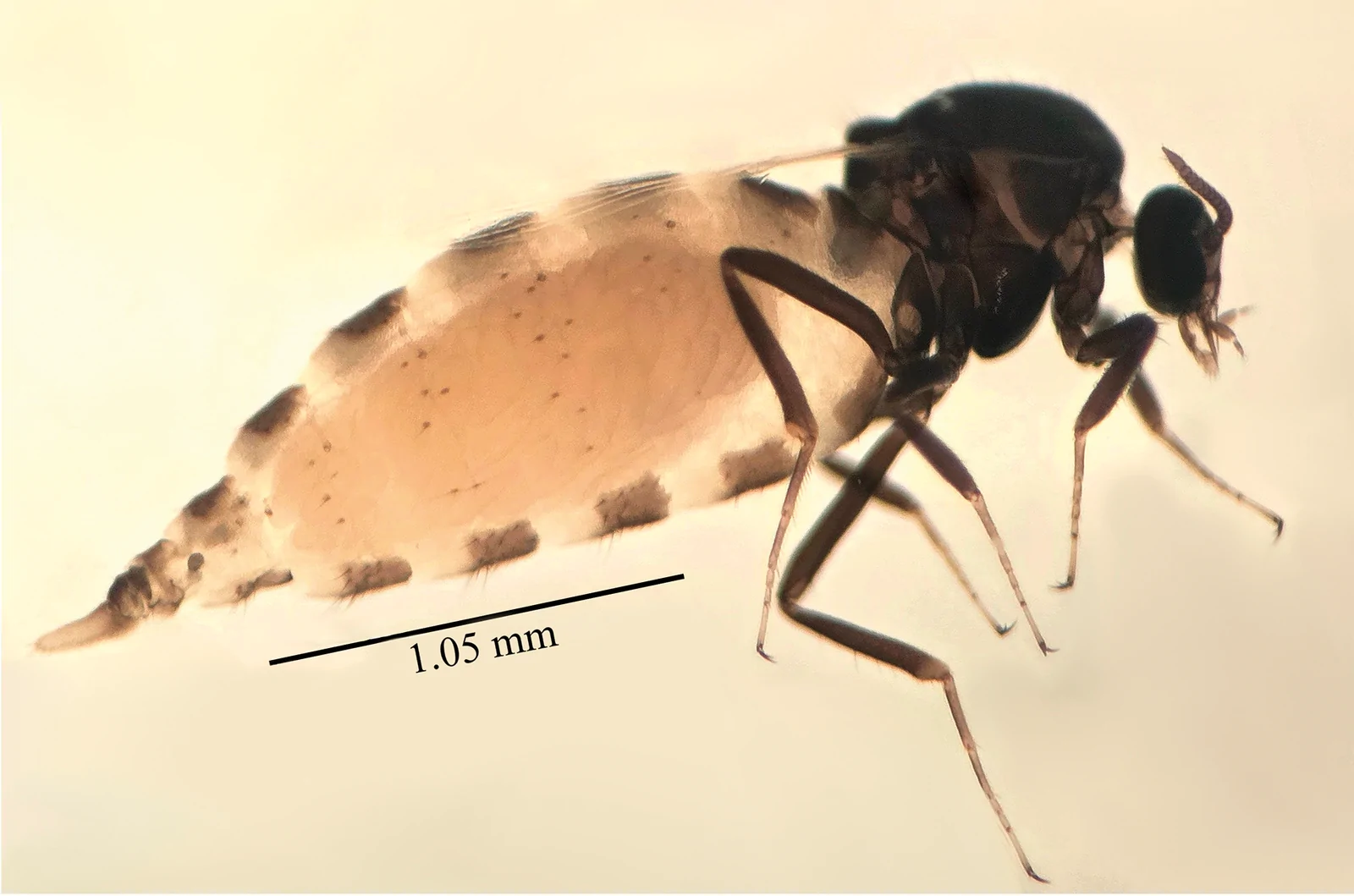
*Leptoconops* (*Megaconops*) *floridensis* collected from the air sacs of a kelp gull (*Larus dominicanus*). Gravid female showing an abdomen full of eggs.

**Fig. 7:**
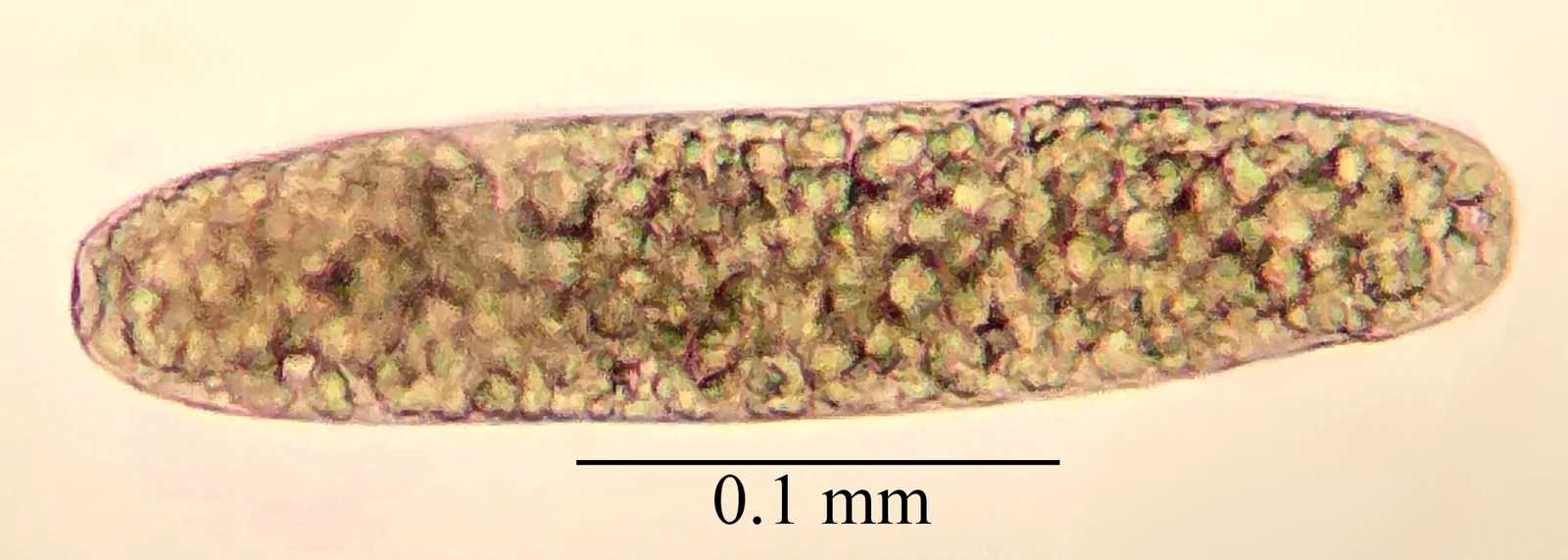
*Leptoconops* (*Megaconops*) *floridensis* egg. Photomicrograph (100×).

*Identification of Ceratopogonidae species* - All 535 specimens collected were female, of which 531 (99.3%) were identified as *Le.* (*M.*) *floridensis* and four (0.7%) as *Culicoides* belonging to two species, *C*. (*Hoffmania*) *diffusus* Spinelli (n = 3 specimens) and *C*. (*Hoffmania*) *brasilianum* Forattini (n = 1 specimen). We observed specimens at various stages of engorgement and egg development (Fig. 5). A total of 440 (82.8%) of the *Le.* (*M.*) *floridensis* collected were gravid or engorged (Figs 5-6), and both species of *Culicoides* were engorged. The eggs taken from gravid females measured approximately 0.3 mm (Fig. 7).

## DISCUSSION

This is the first description of haematophagous Diptera feeding on the internal organs of a vertebrate. These records document a large number of biting midges (n = 535) in the respiratory system of 34 birds, particularly in the trachea, air sacs, and lungs, with more than 51 midges found in five of them. Hematophagy was observed during necropsy, along with inflammation spots suggesting additional antemortem bite sites. In general, insects found in the lungs were dead, either encased in secretion or encapsulated. The discovery of a large number of gravid or engorged *Le.* (*M*.) *floridensis* females in the respiratory system of birds, especially in kelp gulls, with no eggs or larvae observed, indicates that this host serves as an important blood source for reproduction of this biting midge species.

Rescued birds infested by ceratopogonids commonly exhibited dyspnoea, and this condition may have facilitated insect entry, with the insects possibly present in the microhabitat formed by debris deposited on beaches. Respiratory distress/dyspnoea — characterised by increased respiratory rate and effort, open-beak breathing, with or without abnormal respiratory sounds^36^ — can expose the airways and facilitate insect entry, further compromising respiratory function. The trachea and lungs are highly vascularised, resulting in a more effective inflammatory response; indeed, in these sites, many insects were found dead, either encased in secretion or encapsulated. Live insects were more abundant in the air sacs, a site with poor vascularisation and, consequently, a slower inflammatory response.^6^ [Bibr B37] ^37^ However, we were unable to determine how long the insects survived within the avian respiratory system; some birds expelled live insects even after up to five days in captivity.

Most birds (> 60%) were found on beaches located in the access channel region of Babitonga Bay, within estuarine waters (Fig. 1). Beaches in this region consistently receive allochthonous organic material from continental drainage, which is deposited on the sandy shore, forming microhabitats. In tropical and subtropical regions such as Babitonga Bay, these organic deposits may consist of mangrove propagules and animal carcasses,^38^ as well as plant material of varied origin, food scraps, and solid waste. This debris provides a food source and shelter for various invertebrate species, and is colonised by crustaceans (amphipods and isopods) and insects (adults and larvae), particularly dipterans and coleopterans.^39^ Unfortunately, research on the invertebrate fauna associated with organic deposits in beach environments remains scarce, and insects are a highly neglected taxonomic group; further studies are therefore needed to understand these environments as a breeding place for biting midges.

The kelp gull is a seabird species with an opportunistic, generalist diet, commonly observed foraging on waste left on beaches and on fishing discards.^40^ [Bibr B41] ^41^ [Bibr B42] ^42^ This behaviour makes it more susceptible to intoxication, particularly during the warmer seasons (spring and summer), when tourist numbers and waste increase, coinciding with a higher number of records of debilitated kelp gulls.^43^ According to Chupil et al.,^44^ intoxication is among the main causes of weakness and mortality in kelp gulls rescued along the coast of the Santos Basin. In addition to ceratopogonid infestation in the respiratory system, all birds analysed in this study exhibited at least one prior disorder leading to weakness, impaired mobility, and dyspnoea, whether due to respiratory infections, intoxication, or other factors associated with high spring/summer temperatures. Approximately 64% of the kelp gulls and 75% of the neotropic cormorants recorded in the region were thin or emaciated (Table), indicating the chronic nature of these conditions.

Notably, in addition to the infested kelp gulls and neotropic cormorants, another 505 debilitated live seabirds of 26 species stranded alive and debilitated in the study area (coastal and oceanic habitats, both resident and migratory)^43^ [Bibr B45] ^45^ received the same care but did not present ceratopogonids. For the kelp gull in particular, this interaction was not a sporadic occurrence; it suggests an established ecological relationship, observed over four consecutive years, with higher records during the spring and summer seasons.

The spring and summer seasons offer favourable conditions for ceratopogonid reproduction, with higher temperatures and precipitation in the region.^29^ This is also the breeding season for kelp gulls along the northern coast of Santa Catarina, where they form colonies on the Graças and Tamboretes archipelagos.^46^ The reproductive period represents a time of high energy expenditure for birds, requiring intense parental care, mainly to feed their chicks, which may extend for months.^47^ This increased food demand may lead to greater risk exposure, making the species even more opportunistic and susceptible to illness or injury.

In this study, *Le.* (*M.*) *floridensis* was observed in 33 birds of two species (*L. dominicanus* and *N. brasilianum*) (Table), and the two species of *Culicoides* were collected from only one kelp gull. The presence of *Le.* (*M.*) *floridensis* was first recorded in Brazil by Santarém et al.,^28^ using the same samples collected for the present study, on the northern coast of Santa Catarina. Until then, its distribution was considered restricted to Florida (USA), Jamaica, and Colombia.^48^ In both Florida and Colombia, few specimens were collected biting humans, while in Jamaica only one male was collected by light trap. Notably, in Colombia, specimens have been found in coastal areas and near mangroves, which corroborates the abundance of specimens collected in the present study along the coast of São Francisco do Sul and Itapoá, Santa Catarina, Brazil.

The subgenus *Megaconops* is monotypic, being represented solely by *Le. (M.) floridensis*. Its mandible has a sharp-pointed tip without apparent teeth, which makes it notably difficult to insert the proboscis into the host's skin for blood feeding — contributing to the limited records of this species biting vertebrate hosts, including humans. The finding of *Le.* (*M*.) *floridensis* feeding in large numbers within the avian respiratory system suggests an evolutionary adaptation in the blood-feeding behaviour of these insects, whose sharp, toothless mandibles are effective in obtaining blood from highly vascularised internal organs with delicate walls.

*Culicoides* (*Hoffmania*) *diffusus* was described by Spinelli et al.^49^ from the State of Espírito Santo, southeastern Brazil, and is reported here for the first time from the coast of Santa Catarina. *Culicoides* (*Hoffmania*) *brasilianum* is widely distributed throughout the country.^27^ In the present study, these species were found in small numbers, feeding in the oral cavity of kelp gulls, suggesting opportunistic behaviour owing to the debilitated state of the rescued birds, whose respiratory movements facilitated insect entry. The intense growth of the human population and the consequent habitat fragmentation may create new ecological scenarios, in which previously insignificant pathogens begin to cause clinical disease and affect their host populations. Seabirds can be considered sentinels of marine ecosystem health, as they may indicate ecosystem changes through alterations in their behaviour, feeding habits, and reproductive traits.^50^ [Bibr B51] ^51^ Such a scenario underscores the need for research on infectious and non-infectious diseases affecting free-ranging wildlife. Assessing the overall health status of debilitated wild animals, combined with necropsy of deceased individuals, is an essential tool for understanding the ecological relationships between species and their pathogens, helping to detect risks to wild, domestic, and human populations.^52^

## SUPPLEMENTARY MATERIALS

Supplementary material

## Data Availability

The contents underlying the research text are included in the manuscript.

## References

[1] Schuster R, Krone O (2016). Infectious diseases: Parasites. Avian Medicine.

[B2] Svobodová M, Dolnik OV, Čepička I (2017). Biting midges (Ceratopogonidae) as vectors of avian trypanosomes. Parasit Vectors.

[B3] Stacy N, Pendl H (2016). Advances in clinical pathology and diagnostic medicine: cytology. Current therapy in avian medicine and surgery.

[4] Heard DJ (1997). Avian respiratory anatomy. Semin Avian Exot Pet Med.

[B5] O'Malley B (2005). Avian anatomy and physiology. Clinical Anatomy and Physiology of Exotic Species.

[B6] Powell FL (2015). Respiration. Sturkie's Avian Physiology.

[7] Graells G, Celís-Diez JL, Corcoran D, Gelcich S (2022). Bird communities in coastal areas. effects of anthropogenic influences and distance from the coast. Front Ecol Evol.

[B8] Matos AMRN, Domit C, Bracarense APFRL (2020). Seabirds: studies with parasitofauna and potential indicators for environmental anthropogenic impacts. Semina: Ciências Agrárias.

[9] Schlatter RP, Simeone A (1999). Estado del conocimiento y conservación de las aves en mares Chilenos. Estud Oceanol.

[10] Naka LN, Rodrigues M (2000). As aves da ilha de Santa Catarina.

[B11] Branco JO (2000). Avifauna associada ao estuário do Saco da Fazenda, Itajaí, Santa Catarina. Rev Bras Zool.

[B12] Schiefler AF, Soares M (1994). Estudo comparativo da avifauna das praias de Navegantes e Laguna, Santa Catarina. Biotemas.

[13] Miotto LM, Carvalho MB, Spach LH, Barbieri E (2017). Ictiofauna demersal na alimentação do gaivotão (*Larus dominicanus*) em um ambiente subtropical. NOS.

[B14] Branco JO (1999). Biologia do Xiphopenaeus kroyeri (Heller, 1862) (Decapoda: Penaeidae), análise da fauna acompanhante e das aves marinhas relacionadas a sua pesca, na região de Penha, Santa Catarina, Brasil. [PhD Thesis].

[B15] Sick H (1997). Introdução a ornitologia brasileira.

[B16] Bertellot M, Yorio P (2000). Age-related feeding behaviour and foraging efficiency in Kelp Gulls *Larus dominicanus* attending coastal trawlers in Argentina. Ardea.

[B17] Quintana F, Yorio P, García Borboroglu P (2002). Aspects of the breeding biology of the neotropic cormorant *Phalacrocorax olivaceus* at Golfo San Jorge, Patagonia, Argentina. Mar Ornithol.

[B18] Orta J (1992). Handbook of the birds of the world. Vol. 1. Ostrich to ducks.

[B19] Pimenta VRA, Rodrigues-Filho JL, Ataides JS, Branco JO (2024). Human-wildlife conflict between Neotropic cormorant and artisanal fisheries induces dubious management policies in southern Brazil. Ocean Coast Res.

[20] Alarcón PAE, Macchi PJ, Trejo A, Alonso MF (2012). Diet of the Neotropic cormorant (*Phalacrocorax brasilianus*) in a Patagonian freshwater environment invaded by exotic fish. Waterbirds.

[B21] Petracci PF, Cereghetti J, Martín J, Obed YS (2009). Dieta del biguá (*Phalacrocorax olivaceus*) durante la primavera en el estuario de Bahía Blanca, Buenos Aires, Argentina. Hornero.

[B22] Barquete V, Bugoni L, Vooren CM (2008). Diet of Neotropic cormorant (*Phalacrocorax brasilianus*) in an estuarine environment. Mar Biol.

[23] Downes JA, Wirth WW (1982). Manual of Nearctic Diptera. Vol. 1.

[24] Kettle DS (1962). The bionomics and control of *Culicoides* and *Leptoconops* (Diptera, Ceratopogonidae = Heleidae). Annu Rev Entomol.

[25] Lutz A (1913). Contribuição para o estudo das Ceratopogoninas hematofagas do Brasil. Mem Inst Oswaldo Cruz.

[26] Clastrier J, Wirth WW (1978). The *Leptoconops kerteszi* complex in North America (Diptera: Ceratopogonidae). Technical bulletin (United States. Department of Agriculture).

[27] Santarém MCA, Felippe-Bauer ML (2024). Analysis of Brazilian Ceratopogonidae (Diptera: Culicomorpha) species diversity and knowledge assessment. Zoologia.

[28] Santarém MCA, Cremer MJ, Vieira JV, Lemos GG, Pecor DB, Felippe-Bauer ML (2023). Redescription of *Leptoconops (Leptoconops) brasiliensis* (Lutz) and *Leptoconops (Megaconops) floridensis* Wirth (Diptera: Ceratopogonidae) with its first Brazilian record. Zootaxa.

[29] Koehntopp PI, Mello YR, Magna DJ, Simm M, Baldo W, Vieira CV (2021). Caracterização climatológica na Baía Babitonga - parâmetros climáticos e meteorológicos - pluviosidade, temperatura e ventos. Revista CEPSUL - Biodiversidade e Conservação Marinha.

[30] Koehntopp PI (2010). Governança e mudança climática nas cidades contemporâneas o caso de Joinville, Santa Catarina. [PhD Thesis].

[31] MMA/IBAMA - Ministério do Meio Ambiente/Instituto Brasileiro de Meio Ambiente (2007). Reserva de fauna Baía da Babitonga.

[32] Abreu JJ (2011). Transporte sedimentar longitudinal e morfodinâmica praial: exemplo do litoral norte de Santa Catarina. [PhD Thesis].

[33] CFMV - Conselho Federal De Medicina Veterinária (2013). Guia Brasileiro de Boas Práticas em Eutanásia em Animais - Conceitos e Procedimentos Recomendados.

[34] Harrison G, Ritchie BW (1994). Avian medicine: principles and applications. Making distinctions in the physical examination.

[35] Wirth WW, Marston NA (1968). method for mounting small insects on microscope slides in Canada balsam. Ann Entomol Soc Am.

[36] Orosz SE, Lichtenberger M (2011). Avian respiratory distress: etiology, diagnosis and treatment. Vet Cin North Am Exot Anim Pact.

[B37] Xavier MO, Madrid IM (2017). Tratado de animais selvagens: medicina veterinária.

[38] Colombini I, Chelazzi L (2003). Influence of marine allochthonous input on sandy beach assemblages. Ocean Mar Biol: an Annual Review.

[39] Ruiz-Delgado MC, Vieira JV, Veloso VG, Reyes-Martínez MJ, Sallorenzo AI, Borzone CA (2014). The role of wrack deposits for supralittoral arthropods: an example using Atlantic sandy beaches of Brazil and Spain. Estuar Coast Shelf Sci.

[40] Oro D, Genovart M, Tavecchia G, Fowler MS, Martínez-Abraín A (2013). Ecological and evolutionary implications of food subsidies from humans. Ecol Lett.

[B41] Giaccardi M, Yorio P (2004). Temporal patterns of abundance and waste use by Kelp Gulls (*Larus dominicanus*) at an urban and fish waste site in northern coastal Patagonia, Argentina. Ornitol Neotrop.

[B42] Giaccardi M, Yorio P, Lizurume E (1997). Patrones estacionales de la gaivota cocinera (*L. dominicanus*) em un basural Patagónico y sus relaciones con el manejo de residuos urbanos y pesqueiros. Ornitol Neotrop.

[43] SIMBA - Sistema De Informação De Monitoramento Da Biota Aquática https://simba.petrobras.com.br.

[44] Chupil H, Farah RF, Maranho A, Barbosa CB, Leonardi S, Cabral J (2024). Insights into the ecology and conservation of coastal Brazil seabirds based on band returns. Mar Ornithol.

[B45] Barreto AS, Cabral A, Castilho PV, Dicky J, Domit C, Groch KR (2023). Stranded marine mammals, sea turtles and seabirds in Paraná and Santa Catarina from August 2018 to August 2023. Dataset. Dryad.

[46] Grose A V, Fink D, Cremer MJ (2019). Revisão bibliométrica de estudos de avifauna no Ecossistema Babitonga, Santa Catarina, Brasil. Revista CEPSUL - Biodiversidade e Conservação Marinha.

[47] Nord A, Williams JB (2015). Nests, eggs, and incubation: new ideas about avian reproduction.

[48] Wirth WW, Atchley WR (1973). A review of the North American *Leptoconops* (Diptera: Ceratopogonidae). Graduate Studies Texas Tech University.

[49] Spinelli GR, Greiner EC, Wirth WW (1993). The Neotropical bloodsucking midges of the *Culicoides guttatus* group of the subgenus *Hoffmania* (Diptera: Ceratopogonidae). Contrib Am Entomol Inst.

[50] Boersma PD, Clark JA, Hillgarth N (2001). Biology of Marine Birds.

[B51] Furness RW, Monaghan P (1987). Seabird ecology.

[52] Deem SL, Karesh WB, Weisman W (2001). Putting theory into practice: wildlife health in conservation. Conserv Biol.

