# Supplementary material for: A new look at the feeding behaviour of haematophagous Diptera: the biting habits of Leptoconops (Megaconops) floridensis Wirth (Diptera: Ceratopogonidae)
Source: Mem Inst Oswaldo Cruz. 2026 Sep 28;121:e260021. doi: 10.1590/0074-02760260021 (PMC13619161; doi:10.1590/0074-02760260021)
Supplement: Supplementary file 1 [file 1678-8060-mioc-121-e260021-s1.pdf]

Mem Inst Oswaldo Cruz, Rio de Janeiro, Vol. *121*: e260021, 2026

VIDEO

click here to access

The invasive haematophagous behaviour of biting midges within the respiratory tract of seagulls in Santa Catarina, Brazil.

SUPPLEMENTARY DATA
