# Supplementary material for: A new look at the feeding behaviour of haematophagous Diptera: the biting habits of Leptoconops (Megaconops) floridensis Wirth (Diptera: Ceratopogonidae)
Source: Mem Inst Oswaldo Cruz. 2026 Sep 28;121:e260021. doi: 10.1590/0074-02760260021 (PMC13619161; doi:10.1590/0074-02760260021)
Supplement: Peer review history

# Reviewer report: FIRST REVIEW ROUND - REVIEWERS' COMMENTS

## REVIEWER #1

Contains adequate abstract. Originality and relevance the authors propose that these insects may be engaging in hematophagy within internal host tissues, a behavior not previously described for Ceratopogonidae. The Methodology is adequate, Nannopterum brasilianum vs brasillianum (review name) Laurus dominicanus (Larus) Review name results, discussion, references and figures and tables are adequate.

## REVIEWER #2

The manuscript is very interesting. This is the first recorded occurrence of ceratopogonids feeding in the innermost part of the respiratory system of birds and that biting midges habits may cause morbidity and mortality to these animals. I have just minor suggestions. Mato Grosso also had occurrence of Leptoconops (see ttps://cms.ufmt.br/files/galleries/95/Led432470115768072dd501a6ffc90a2c7ea9b0ab.pdf). For restinga ecosystem, maybe could be interesting add a small explanation in english- sandbank, spit, ou coastal sand dune vegetation. Is necessary CEUA for Euthanasia in the case of that birds? I did not find the Supplemental material for the video showing the ceratopogonids on respiratory system of Larus dominicanus. The first paragraph of discussion is not necessary.

## AUTHORS' RESPONSE TO THE REVIEWERS

Dear. Dr. Adeilton Brandão,

Thank you for your kind reply.

Reviewer 1:

-The names were corrected. I have marked them in green.

Thank you for the valuable suggestions for this study.

Reviewer 2:

The corrections were made and marked in yellow.

- In agreement with Reviewer, we removed the first paragraph from the Discussion.

- An ethical approval statement does not apply to this study, as all clinical procedures were conducted as part of routine veterinary care. I have inserted this explanation on the text. Thank you for pointing this.

- In agreement with Reviewer, "coastal sand dune vegetation" was added to explain "restinga".

- The Introduction reports the three species recorded in Brazil and the states where they were found. Since the manuscript addresses the observation of Leptoconops floridensis feeding on the respiratory system of birds along the coast of Santa Catarina, we did not find it pertinent to include the record of Leptoconops specimens from the state of Mato Grosso, as it is not a species-specific record. We thank the reviewer for the literature suggestion, which will be valuable for future studies on the distribution of the genus Leptoconops in Brazil.

-I've followed the instructions to upload the video as a "supplementary file for review" both online and offline. Check if you can access the video now (filme ceratopogonidae em Larus dominicanus.mp4). If you don't, please let me know, and then I can contact the editor for additional support.

Thank you for all the valuable suggestions for this study.

# Reviewer report: REVIEWERS' COMMENTS

## REVIEWER #1

I kept my comments on the first review. The response of the authors, all items are ok for me.

## REVIEWER #2

The manuscript presents a highly novel and relevant observation regarding the invasive hematophagous behavior of Leptoconops (Megaconops) floridensis within the respiratory tract of seabirds. The study is scientifically valuable and supported by an important number of specimens and well-conducted taxonomic identification.

The work may represent a significant contribution to medical and veterinary entomology.

DECISION: ACCEPT SUBMISSION | RECEIVED 24 JANUARY 2026 | ACCEPTED 29 MAY 2026
